# Immunogenicity of standard, high-dose, MF59-adjuvanted, and recombinant-HA seasonal influenza vaccination in older adults

**DOI:** 10.1038/s41541-021-00289-5

**Published:** 2021-02-16

**Authors:** Athena P. Y. Li, Carolyn A. Cohen, Nancy H. L. Leung, Vicky J. Fang, Shivaprakash Gangappa, Suryaprakash Sambhara, Min Z. Levine, A. Danielle Iuliano, Ranawaka A. P. M. Perera, Dennis K. M. Ip, J. S. Malik Peiris, Mark G. Thompson, Benjamin J. Cowling, Sophie A. Valkenburg

**Affiliations:** 1 HKU-Pasteur Research Pole, School of Public Health, The University of Hong Kong, Hong Kong SAR, China; 2 WHO Collaborating Centre for Infectious Disease Epidemiology and Control, School of Public Health, The University of Hong Kong, Hong Kong SAR, China; 3 Influenza Division, Centers for Disease Control and Prevention, Atlanta, GA USA

**Keywords:** Antibodies, Adjuvants, Immunological memory

## Abstract

The vaccine efficacy of standard-dose seasonal inactivated influenza vaccines (S-IIV) can be improved by the use of vaccines with higher antigen content or adjuvants. We conducted a randomized controlled trial in older adults to compare cellular and antibody responses of S-IIV versus enhanced vaccines (eIIV): MF59-adjuvanted (A-eIIV), high-dose (H-eIIV), and recombinant-hemagglutinin (HA) (R-eIIV). All vaccines induced comparable H3-HA-specific IgG and elevated antibody-dependent cellular cytotoxicity (ADCC) activity at day 30 post vaccination. H3-HA-specific ADCC responses were greatest following H-eIIV. Only A-eIIV increased H3-HA-IgG avidity, HA-stalk IgG and ADCC activity. eIIVs also increased polyfunctional CD4+ and CD8+ T cell responses, while cellular immune responses were skewed toward single-cytokine-producing T cells among S-IIV subjects. Our study provides further immunological evidence for the preferential use of eIIVs in older adults as each vaccine platform had an advantage over the standard-dose vaccine in terms of NK cell activation, HA-stalk antibodies, and T cell responses.

## Introduction

Older adults (≥65 years) account for the majority of influenza-related morbidity and mortality each year^1^ and are considered a priority group for annual vaccination. Immunization with the standard-dose seasonal inactivated influenza vaccine (S-IIV) remains the most effective public health intervention against infection by seasonal influenza A and B viruses for older adults. Yet, vaccine effectiveness can be lower in older adults compared to younger age groups^2^. Enhanced inactivated influenza vaccines (eIIV) that induce greater hemagglutinin inhibition (HAI) titers to the immunodominant surface HA glycoprotein and confer superior immunogenicity and/or vaccine efficacy compared to S-IIV in preventing influenza-related medical complications have recently become available^3–5^. These eIIVs, include Fluad (MF59-adjuvanted IIV, A-eIIV), fluzone-high-dose (IIV, H-eIIV), and Flublok (recombinant-HA IIV, R-eIIV).

Fluad contains the MF59 adjuvant, which is an oil-in-water emulsion of squalene. MF59-adjuvanted influenza vaccines have been shown to boost IFN-γ^+^ T cells^6^ and CD4^+^ T cell helper activity^7^. MF59 is also a potent inducer of germinal center (GC) reactions and increases the magnitude, diversity, affinity^8^, and persistence of influenza virus-specific antibodies. Fluzone-high-dose contains four times the amount of HA protein than standard-dose S-IIV. The benefit of high-dose over S-IIV varies depending on seasonal strain dominance and appears strongest during A(H3N2) dominant seasons^9^. In older adults, this vaccine stimulates greater T follicular helper (TFH) cell activation and plasmablast recruitment^10^, while IFN-γ^+^ T cell responses are boosted but comparable to S-IIV^11^. Flublok contains three times the amount of HA as S-IIV. Although not originally designed solely for older populations, Flublok provided improved protection against laboratory-confirmed infection in older adults during a A(H3N2) dominant season despite an antigenic mismatch between the vaccine and circulating A(H3N2) strain^4^. As a recombinant protein vaccine produced via insect cell culture-based baculovirus expression systems, Flublok also has an advantage in that it can be made without egg-based adaptations.

Antibodies measured by the HAI assay have traditionally been used as the gold standard of vaccine-induced correlate of protection (CoP)^12^ despite representing only a fraction of the total immune response to vaccination. Instead, protection likely requires a multi-pronged immune response involving a range of humoral and cellular mechanisms that cannot be assessed alone by traditional HAI assays. Furthermore, influenza infection may occur in individuals despite high HAI responses following vaccination or infection^13^, or in some cases, individuals may not become seropositive (HAI titer ≥ 1:40)^14^. Universal influenza vaccine development is currently a global priority to improve protection against diverse influenza virus strains and across all age groups. This will first require the identification of additional CoPs with known clinical efficacy. Hence, a deeper understanding of how currently available vaccines stimulate multiple arms of the immune system is necessary.

Recently, advances have been made in identifying additional CoPs. TFH cells have been shown to play a crucial role in the generation of antibody responses following S-IIV vaccination^15^ and age-related impairment in the recruitment and helper capacity of TFH cells have been associated with suboptimal antibody responses in older adults^16^. Antibody-dependent cellular cytotoxicity (ADCC) and memory T cell responses have been associated with protection against symptomatic infection^17–19^ and may represent additional correlates of protection in older adults. Meanwhile, there remains unresolved concerns for immune interference by prior or repeat vaccination and infection on current season vaccine effectiveness based on observational studies^20^, which is most relevant to older adults who may typically receive multiple consecutive vaccinations.

We conducted a randomized controlled trial to compare the immunogenicity of three vaccines, Fluad, fluzone-high-dose, and Flublok versus standard Fluquadri (S-IIV)^21^. We previously reported that all three vaccines induced greater day 30 HAI titers and mean fold rise of HAI antibodies against A(H3N2) vaccine-matched, egg-derived virus compared to S-IIV^21^. R-eIIV was superior when neutralizing antibody titers were assessed using cell-derived A(H3N2) virus, followed by H-eIIV and A-eIIV, and all eIIVs stimulated some boosting of T cell responses to multiple influenza virus strains. In this current study, we further analyzed a subset of participants to determine whether improved immunogenicity following enhanced vaccination may be attributable to other humoral and cellular parameters besides HAI. Since the A(H3N2) virus undergoes antigenic drift more rapidly than A(H1N1) virus subtypes, it poses a more significant hurdle for vaccine-mediated protection^22^ and we, therefore, focused our study on assessing responses against A(H3N2).

For a more comprehensive insight into humoral immune responses, we measured the magnitude of antibody responses directed against HA protein as well as the quality of response by assessing polyclonal antibody avidity, stalk-binding activity, and IgG subclass distribution related to Fc receptor-mediated (FcR) effector functions. We also measured the recruitment of CD4^+^ TFH cells and multiple-cytokine-secreting CD4^+^ and CD8^+^ T cells to study the interplay between humoral and cellular responses. These immune parameters were selected due to their contribution in mediating the immune response against the influenza virus as well as their relevance for universal vaccine development. Finally, we explored whether enhanced vaccination has the potential to revive immune responses among individuals with extensive prior vaccination history.

## Results

### Study design and population

**Fig. 1:**
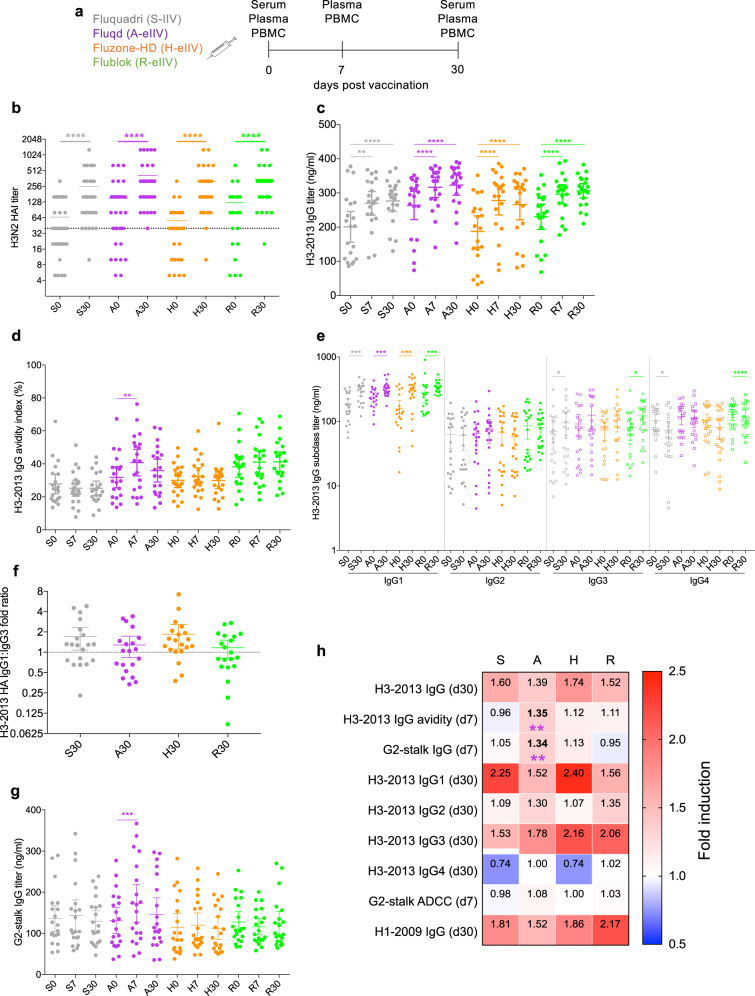
Analysis of antibody responses against hemagglutinin and G2-stalk proteins. **a** Subjects were randomized to receive either Fluquadri, Fluad, Fluzone-High-Dose, or Flublok. Serum, plasma, and PBMCs were collected at various time-points. **b** A(H3N2)-2014 serum HAI antibody titers (S, *n* = 37; A, *n* = 34; H, *n* = 30; R, *n* = 23). **c** H3-2013 HA-specific IgG antibody titers (*n* = 20 per vaccine group). **d** Proportion of high-avidity H3-2013 HA-specific IgG. **e** H3-2013 HA-IgG subclass titers and **f** ratio between the fold expansion of IgG1 versus IgG3 at day 30 post vaccination. **g** G2-stalk-specific IgG titers. **h** Summary heat map depicting mean fold induction of IgG antibody responses from (**b**–**g**) and H1-2009 HA (from Supplementary Fig. 3a). Wilcoxon signed-ranked test for two-group comparisons. For multiple-group comparisons, Friedman’s test for within-group (colored*) and Kruskal–Wallis test for between-group (black*) comparison was performed. *p < 0.05, **p < 0.01, ***p < 0.001, ****p < 0.0001 versus day 0 or S-IIV, as indicated. Data represented as mean with 95% CI; each dot represents a single individual. S standard (S-IIV), A adjuvanted (A-eIIV), H high-dose (H-eIIV), R recombinant (R-eIIV).

We examined antibody and cellular responses following S-IIV (*n* = 37), A-eIIV (*n* = 34), H-eIIV (*n* = 30), and R-eIIV (*n* = 23) (Fig. 1a) vaccination in 124 older adults (Table 1) randomly selected per group from an initial total cohort of 800 subjects^21^ recruited from October 2017 to January 2018. Participants did not differ significantly in age, sex, prior vaccination history, and underlying chronic medical conditions or smoking status (Table 1). Self-reported or clinically recorded influenza vaccination status in the past 5 years showed that 60% of participants had received trivalent S-IIV influenza vaccination in the previous 2016/2017 season. Serological (*n* = 20–37 per group) and cellular assays (*n* = 13–24 per group) were performed in parallel for most participants, where matched specimens and complete time-points were available (Table 2).

**Table 1:** Baseline characteristics of participants, by vaccination group.

Characteristic	Vaccination group				
	S-IIV (Fluquadri) (*n* = 37)	A-eIIV (Fluad) (*n* = 34)	H-eIIV (Fluzone-high dose) (*n* = 30)	R-eIIV (Flublok) (*n* = 23)	
	*n* (%)	*n* (%)	*n* (%)	*n* (%)	*p* Value
*Age, years*					
65–70	25 (67.6%)	18 (52.9%)	24 (80.0%)	12 (52.2%)	0.173
71–76	6 (16.2%)	11 (32.4%)	4 (13.3%)	5 (21.7%)	
77–82	6 (16.2%)	5 (14.7%)	2 (6.7%)	6 (26.1%)	
Female gender	21 (57%)	22 (65%)	22 (73%)	12 (52%)	0.52
Underlying medical conditions^a^	27 (73%)	29 (85%)	21 (70%)	21 (91%)	0.16
Actively smoking	3 (8.1%)	3 (8.8%)	3 (10.0%)	1 (4.4%)	
Received influenza vaccination in 2016/2017 season	23 (62%)	17 (50%)	20 (67%)	15 (65%)	0.89
At least one prior vaccination in past 5 years^b^	28 (76%)	24 (71%)	22 (73%)	16 (70%)	0.95
“Regularly immunized” (≥3 vaccinations in the past 5 years) ^b^	18 (49%)	18 (53%)	11 (37%)	10 (43%)	0.60
“Not regularly immunized” (≤2 vaccinations in the past 5 years)^b^	19 (51%)	16 (47%)	19 (63%)	13 (57%)	
Seronegative at baseline^c^	16 (43%)	8 (23%)	12 (40%)	6 (26%)	0.24
Seropositive at baseline^d^	21 (57%)	26 (76%)	18 (60%)	17 (74%)	
Seropositive post vaccination^e^	36 (97%)	34 (100%)	29 (97%)	22 (96%)	0.72
Seroconverted post vaccination (≥4-fold increase)^f^	17 (46%)	14 (41%)	20 (67%)	9 (39%)	0.13

*Note*: Northern Hemisphere 2017/2018 vaccine recommended trivalent strains: A/Michigan/45/2015 (H1N1) pdm09-like virus, A/Hong Kong/4801/2014 (H3N2)-like virus and B/Brisbane/60/2008-like virus (Victoria lineage). Quadrivalent vaccines, included a B/Phuket/3073/2013 strain (Yamagata lineage)

^a^Underlying medical conditions defined as any of the following: hypertension, osteoarthritis, diabetes, heart diseases, cancer, stroke, chronic lung disease, kidney disease, liver disease, depression or anxiety disorder, neurological disorder, autoimmune diseases, gastrointestinal diseases, hypothyroidism, or dermatological diseases.

^b^Including northern hemisphere formulations from 2012/2013 through 2016/2017 and southern hemisphere formulation in 2015.

^c^Seronegative defined as subjects with pre-vaccination (day 0) HAI titer of <40.

^d^Seropositive defined as subjects with pre-vaccination (day 0) HAI titer of ≥40.

^e^Seroprotection defined as subjects with post-vaccination (day 30) HAI titer of ≥40.

^f^Seroconversion defined as subjects with ≥4-fold rise in post-vaccination (day 30) HAI titers if pre-vaccination titer was ≥10, or a post-vaccination HAI titer of ≥40 if pre-vaccination titer was <10.

*p* Values represent the chi-square test for categorical variables; significance is defined at *p* < 0.05.

**Table 2:** Immunological assays with matched humoral and cellular results available.

Immunological assays	Vaccination group			
	S-IIV (Fluquadri) (*n* = 37)	A-eIIV (Fluad) (*n* = 34)	H-eIIV (Fluzone-HD) (*n* = 30)	R-eIIV (Flublok) (*n* = 23)
	*n* (matched with humoral)			
Hemagglutinin inhibition assay	37	34	30	23
ELISA and ADCC	20 (20)	20 (20)	20 (20)	20 (20)
TFH/Plasmablast staining	24 (16)	20 (16)	13 (12)	15 (14)
ICS for T cell polyfunctionality	24 (24)	24 (24)	23 (23)	23 (23)

HAI responses were previously reported^21^ and determined using vaccine-matched, egg-derived A(H3N2) A/Hong Kong/4801/2014 (H3-2014) virus. Subsequent humoral assays were performed using A(H3N2) A/Switzerland/9715293/2013 (H3-2013) HA0 recombinant protein, as only the HA1 domain of the vaccine A/Hong Kong/4801/2014 was commercially available at the time of study onset. H3-2013 was last used during the 2015/2016 vaccination season. Therefore, measurement of H3-2013-specific responses resulting from the 2017/2018 vaccination may also represent back-boosting of 2015/2016 vaccine responses for H3-2013-specific B cells. Nevertheless, the IgG response against both HA proteins was significantly correlated (*R* = 0.64, *p* = 0.0299, Supplementary Fig. 1a). As such, we used H3-2013 for further characterization of vaccine responses with group 2 (G2)-stalk response characterized separately.

### A-eIIV improves antibody avidity and boosts stalk antibodies against H3-2013-HA

We previously found in our initial study that all vaccines resulted in significantly greater HAI titers at day 30^21^. Similarly, we observed significantly increased HAI titers post-vaccination in our subset of participants at day 30 (Fig. 1b). HAI antibodies are a fraction of the total pool of influenza virus-specific antibodies and antibodies that bind outside the globular head domain or have effector functions beyond inhibition of host sialic-binding may exist. Therefore, we measured levels of total H3-2013 HA-specific IgG by ELISA for magnitude, avidity, subclass distribution, and HA-stalk-binding ability. We found that all vaccine recipients had significantly increased IgG titers at days 7 and 30 post vaccination compared to day 0 baseline (Fig. 1c). A stringent urea wash was performed to remove low avidity antibodies and an avidity index representing the remaining proportion of high-avidity antibodies relative to total H3-2013 HA-IgG was determined. Only A-eIIV recipients had significantly increased proportions of high-avidity H3-2013 HA-specific IgG at day 7 compared to day 0 (Fig. 1d).

Antibody-mediated antiviral effector function is determined by a hierarchy of IgG subclasses (IgG1–4), with IgG1 and IgG3 being the predominant mediators of anti-viral effector functions^23^. All four subclasses had detectable H3-2013-specific responses, but only IgG1 and IgG3, and not IgG2 and IgG4, were increased at day 30. Notably, the IgG1 subclass was significantly elevated in response to all four vaccines (Fig. 1e). Whereas for IgG3, there was either a significant rise in S-IIV and R-eIIV or a trend for an increase in A-eIIV and H-eIIV recipients. When comparing the ratio of IgG1:IgG3 fold expansion at day 30, there was greater IgG1 expansion than IgG3 on average across all vaccine groups (Fig. 1f).

We next measured antibody responses directed against the functionally conserved G2-stalk region. Only A-eIIV elicited an early day 7 significant increase in G2 stalk-specific IgG (Fig. 1g), for which the magnitude of response was significantly greater than S-IIV (Fig. 1h). G2-stalk responses in A-eIIV recipients were not enhanced for avidity (Supplementary Fig. 2a) and preferentially boosted for IgG1 subclass rather than IgG3 (Supplementary Fig. 2b, c). Additional influenza A virus vaccine antigens were similarly assessed, with significant increases in H1-2009 HA-specific IgG in all vaccine recipients **(**Supplementary Fig. 3a) and group 1 (G1) stalk-IgG in A-eIIV and H-eIIV recipients (Supplementary Fig. 3b). R-eIIV reduced NP-IgG responses following vaccination (Supplementary Fig. 3c), while N1-2015 IgG was increased after A-eIIV and H-eIIV (Supplementary Fig. 3d) and no differences in N2-2015 IgG was observed between vaccine groups (Supplementary Fig. 3e). Overall, we found that the fold induction of H3-2013 and H1-2009 HA IgG responses was comparable between enhanced and standard vaccines (Fig. 1h). Besides A-eIIV stimulating a greater proportion of high-avidity H3-2013 HA IgG and G2-stalk IgG compared to S-IIV, there were no significant differences in the induction of IgG response against another influenza A virus vaccine antigens between the vaccine groups (Fig. 1h and Supplementary Fig. 3f).

### Effect of enhanced vaccines on NK cell activation against H3-2013-HA and Group 2-stalk

**Fig. 2:**
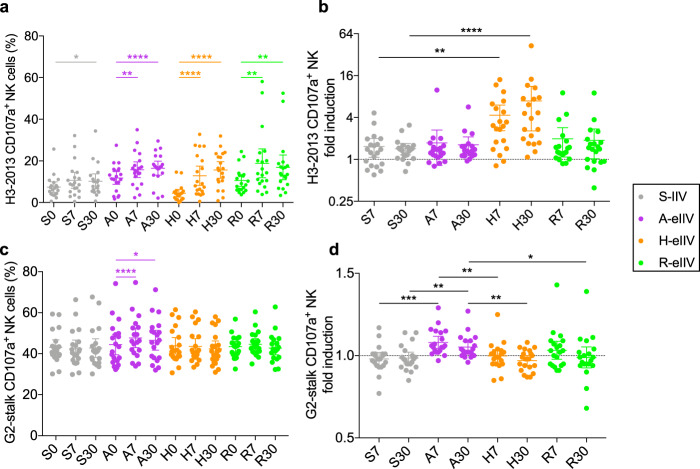
Boosting of ADCC responses by seasonal influenza vaccines against H3-2013 hemagglutinin and G2-stalk protein. Frequency (%) of H3-2013 HA-specific CD107a^+^ NK cells (**a**) and fold induction (**b**). Frequency of G2-stalk-specific CD107a^+^ NK cells (**c**) and fold induction (**d**). Friedman’s test for within-group and Kruskal–Wallis test for between-group comparison was performed. *p < 0.05, **p < 0.01, ***p < 0.001, ****p < 0.0001 versus day 0 or comparison group, as indicated. Data represented as mean (*n* = 20 per vaccine group) with 95% CI.

Antibodies that mediate ADCC can be boosted by seasonal vaccination in older adults^24^ and can be highly cross-reactive between influenza virus subtypes. We measured whether ADCC activating antibodies were boosted by eIIV vaccination by measuring the degranulation of an NK cell line crosslinked by H3-2013 HA-bound antibodies (Supplementary Fig. 4a). All vaccine recipients had significantly increased H3-2013 HA-specific ADCC antibodies by day 30 post vaccination (Fig. 2a), and the fold induction of response was greatest in H-eIIV (6.9-fold) compared to S-IIV recipients (1.4-fold) (Fig. 2b). Notably, increased ADCC antibodies were observed in both A(H3N2) HAI responders (≥4-fold rise seroconversion) and nonresponders (no seroconversion) (Supplementary Fig. 4b), although responders had greater fold induction of responses (Supplementary Fig. 4c). We next evaluated whether G2 stalk-specific ADCC antibodies were boosted, as the HA-stalk epitope is a strong inducer of Fc receptor-mediated effector responses including ADCC^25^. Strikingly, only A-eIIV recipients had significantly increased stalk-specific NK cell degranulation following vaccination (Fig. 2c) and their responses were at a greater magnitude compared to other vaccine types across multiple time points (Fig. 2d). An increase in G2-stalk ADCC antibodies amongst A-eIIV recipients was observed in both HAI responders and nonresponders (Supplementary Fig. 4d) with no differences in the strength of response (Supplementary Fig. 4e).

### Early induction of activated type-1 TFH cells by A-eIIV and H-eIIV predict superior antibody responses

**Fig. 3:**
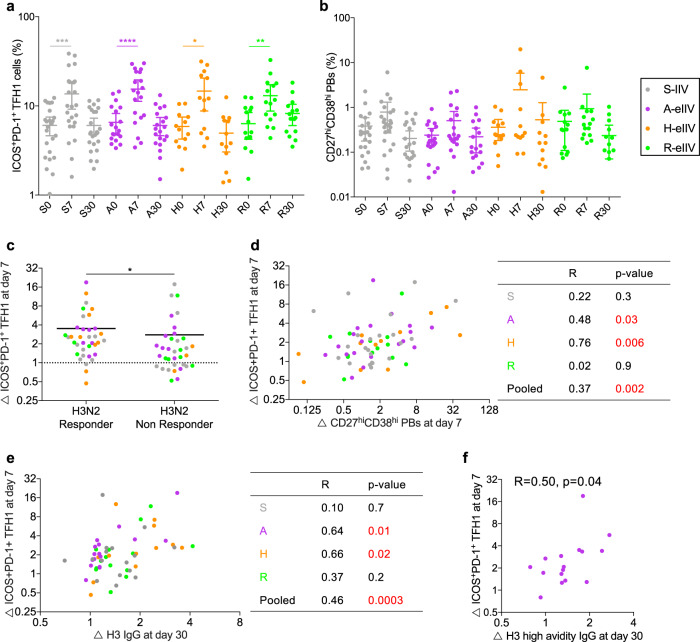
Interplay between CD4^+^ T follicular helper cells and humoral responses. The frequency of **a** activated ICOS^+^PD-1^+^ TFH1 cells and **b** CD27^hi^CD38^hi^ plasmablasts, significant by Friedman’s test. **c** Fold induction of ICOS^+^PD-1^+^ TFH1 cells by A(H3N2) HAI seroconversion status, the significance by Mann–Whitney *t* test. **d** Spearman’s correlation between the fold induction of ICOS^+^PD-1^+^ TFH1 and plasmablasts at day 7, **e** H3-2013 HA-specific IgG and **f** high-avidity H3-2013 HA-specific IgG, at day 30. Data represented as mean (S, *n* = 24; A, *n* = 20; H, *n* = 13; R, *n* = 15) with 95% CI; *p < 0.05, **p < 0.01, ***p < 0.001, ****p < 0.000. TFH1 type-1 T follicular helper, PB plasmablasts.

In healthy adults, influenza vaccination induces a transient increase of circulating TFH cells that express activation markers ICOS and PD-1^15^ (Supplementary Fig. 5a). Among this activated ICOS^+^PD-1^+^ population, the type-1 subset (TFH1) plays a crucial role in helping B cell plasmablasts (CD27^hi^CD38^hi^CD19^+^) (Supplementary Fig. 5b) secrete influenza virus-specific antibodies^15^. Similarly, we observed a significant increase in activated TFH1 cells (ICOS^+^PD1^+^CXCR3^+^CCR6^−^) (Fig. 3a) and a peak in plasmablasts (Fig. 3b) at day 7 post vaccination. However, there were no statistically significant differences in the magnitude of recruitment between vaccine groups.

A(H3N2) HAI responders (≥4-fold rise seroconversion) had significantly higher recruitment of ICOS^+^PD1^+^ TFH1 cells (*p* < 0.05) (Fig. 3c) compared to nonresponders. However, only A-eIIV and H-eIIV recipients effectively recruited these responses, with a significant positive correlation between the rise of ICOS^+^PD1^+^ TFH1 cells and plasmablasts at day 7 (Fig. 3d), and ICOS^+^PD1^+^ TFH1 cells versus IgG (Fig. 3e) titers at day 30. In fact, the association between ICOS^+^PD1^+^ TFH1 recruitment and IgG antibodies was evident as early as day 7 in the A-eIIV group (Supplementary Fig. 5c) and was also associated with the rise in high-avidity IgG titers at day 30 (Fig. 3f).

### A-eIIV and R-eIIV expand recruitment of polyfunctional T cells that are associated with greater HAI responses

**Fig. 4:**
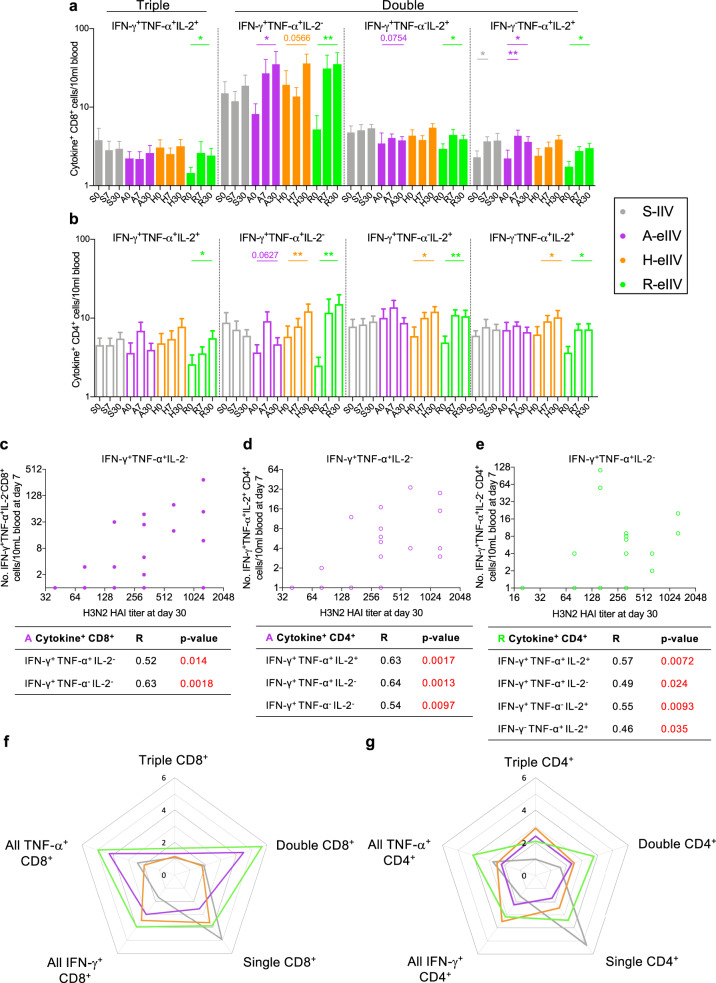
A(H3N2)-specific polyfunctional T cell responses are expanded by enhanced vaccination. The magnitude of multiple-cytokine-secreting **a** CD8^+^ and **b** CD4^+^ T cells at days 0, 7, and 30 post vaccination. Data represented as mean (S, *n* = 24; A, *n* = 24; H, *n* = 23; R, *n* = 23) with SEM, the significance by Friedman’s test. Spearman’s correlation between A(H3N2) HAI titers at day 30 and the recruitment of **c** day 7 cytokine^+^ CD8^+^ subsets in A-eIIV recipients, and **d** cytokine^+^ CD4^+^ subsets in A-eIIV recipients and in **e** R-eIIV recipients. Radar charts depicting mean fold induction of **f** CD8^+^ and **g** CD4^+^ T cell responses at day 7. *p < 0.05, **p < 0.01.

Polyfunctional T cells have been shown to be functionally superior to single-cytokine-producing cells in antiviral immunity^26^ and cytotoxic CD8^+^ T cells correlate with protection from influenza^17^. We, therefore, analyzed the frequencies of single-positive (IFN-γ^+^, TNF-α^+^, and IL-2^+^), double-positive (IFN-γ^+^TNF-α^+^IL-2^−^, IFN-γ^+^TNF-α^-^IL-2^+^, and IFN-γ^−^TNF-α^+^IL-2^+^), and triple-positive (IFN-γ^+^TNF-α^+^IL-2^+^) cytokine-secreting CD4^+^ and CD8^+^ T cells following vaccination (Supplementary Fig. 6a). We found that enhanced vaccines were more capable of stimulating both single- and multiple-cytokine-secreting CD8^+^ T cell responses (Fig. 4a and Supplementary Fig. 6b) in comparison to S-IIV, whereas R-eIIV most robustly stimulated CD4^+^ T cells cytokine secretion (Fig. 4b and Supplementary Fig. 6c). Importantly, there was a significant expansion of double-positive (IFN-γ^+^TNF-α^+^IL-2^−^) CD8^+^ T cells in A-eIIV (*p* = 0.03) and R-eIIV (*p* = 0.0006), a subset of T cells previously described for its importance in reducing disease severity during pandemic H1N1 infection^17^. Moreover, the emergence of double (IFN-γ^+^TNF-α^+^IL-2^−^) and single (IFN-γ^+^TNF-α^−^IL-2^−^) CD8^+^ T cells in the A-eIIV group also positively correlated with HAI titers (Fig. 4c).

Activated and proliferating CD4^+^ T cells are associated with protection from human experimental challenge with influenza A virus^18^. We observed that the emergence of triple, double and single-cytokine-producing CD4^+^ T cells at day 7 were correlated to elevated HAI titers only in A-eIIV (Fig. 4d) and R-eIIV recipients (Fig. 4e), suggesting that better quality cytokine responses early post vaccination possibly contributed to the rise of superior HAI titers in ensuing days. Stratification of cytokine^+^ CD8^+^ (Fig. 4f) and CD4^+^ (Fig. 4g) T cells by polyfunctionality showed that S-IIV skews T cells toward single-cytokine production, whereas enhanced vaccines expanded more differentiated responses including double- and triple-cytokine-secreting T cells.

### Impact of prior influenza vaccination on post-vaccination induction of cellular and H3-2013-specific antibody responses

**Fig. 5:**
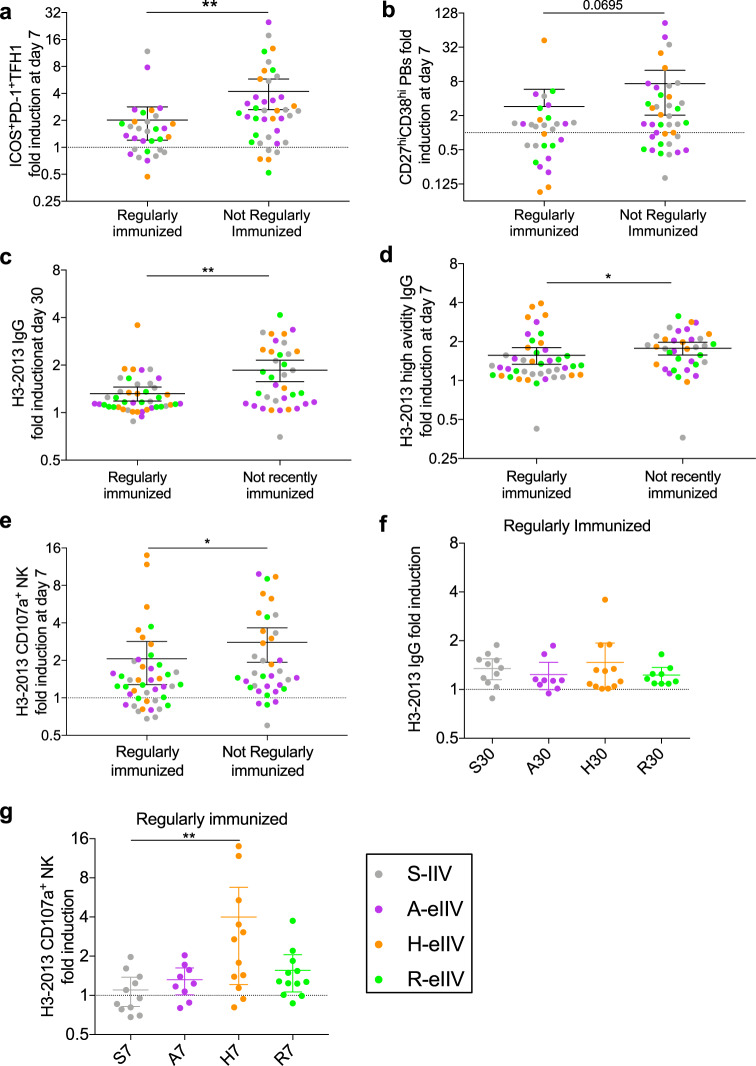
Vaccine responses are restricted by prior vaccination. Subjects were separated by those regularly (≥3 vaccinations) and not regularly immunized (<2 vaccinations) in the past 5 years and the fold induction of **a** TFH1 cells and **b** plasmablasts (S, *n* = 24; A, *n* = 20; H, *n* = 13; R, *n* = 15), **c** H3-2013 HA**-**specific IgG, **d** high-avidity IgG, and **e** CD107a^**+**^CD56^+^ NK cells (*n* = 20 per vaccine group) was determined in pooled datasets, significance by Mann–Whitney *t* test. Fold induction of **f** day 30 H3**-**2013 HA-specific IgG, **g** day 7 NK cell activation amongst regularly immunized subjects (*n* = 9–12 per vaccine group). Data represented as mean with 95% CI. *p < 0.05, **p < 0.01, ***p < 0.001.

Previous studies have reported immune interference from prior or repeat vaccination on current season vaccine immunogenicity of humoral and cellular responses^27^ and we sought to investigate whether receipt of eIIVs can overcome these limitations. Due to a high proportion of older adults having had prior S-IIV vaccinations, subjects were separated into those “regularly immunized” (≥3 vaccinations) or “not regularly immunized” (≤2 vaccinations) in the past 5 years (Table 1). For initial analysis, we pooled eIIV and S-IIV vaccine groups due to limited sample sizes and if significance was observed in aggregate data we further stratified into individual vaccine groups. We found that regularly immunized subjects had significantly lower recruitment of ICOS^+^PD1^+^TFH1 cells compared to those not regularly immunized (Fig. 5a). However, no differences in recruitment of IFN-γ^+^ CD8^+^ and IFN-γ^+^ CD4^+^ T cells were observed (Supplementary Fig. 7a, b). In addition, regularly immunized individuals had a trend for reduced plasmablast induction (*p* = 0.07) (Fig. 5b) and significantly reduced boosting of H3-2013 HA-specific IgG (*p* = 0.004) (Fig. 5c), high-avidity IgG antibodies (*p* = 0.02) (Fig. 5d) and ADCC responses (*p* = 0.03) (Fig. 5e). In contrast, there were no differences in the induction of G2-stalk-specific IgG (Supplementary Fig. 7c) and ADCC responses (*p* = 0.42) (Supplementary Fig. 6d).

There was no notable improvement in the induction of H3-2013 HA-specific IgG responses by eIIVs in regularly immunized subjects at day 30 post vaccination (Fig. 5f). However, amongst more frequently vaccinated individuals, H-eIIV recipients had greater induction of ADCC than S-IIV at day 7 post vaccination (*p* = 0.002) (Fig. 5g).

## Discussion

Multiple enhanced seasonal influenza vaccines are now available for older adults, yet a consensus on the prioritization of a preferred vaccine has not been determined due to a lack of consistent data from different studies^28^. We found that enhanced vaccines consistently outperformed standard-dose vaccination across a range of immune parameters. A lack of confirmed CoPs for influenza virus infection beyond HAI and microneutralization (MN) means that despite our systematic dissection of immunogenicity, we are unable to ascertain a single enhanced vaccine for preferential use in older adults.

We found that A-eIIV stimulated an early (day 7) increase in the proportion of high-avidity antibodies compared to S-IIV but it is not maintained long term, which has also been observed by others^29^. A previous study also observed that an early peak in IgG avidity coincided with the recruitment of activated ICOS^+^PD-1^+^ TFH1 cells leading to rapid recruitment of memory B cells (MBC) within 7 days post vaccination, possibly in extrafollicular sites^30^. In addition, previous studies have shown that TFH1 cells help MBCs but not naïve B cells differentiate into antibody-secreting plasmablasts and plasma cells in vitro^15^ and that plasmablasts induced by influenza vaccination are often derived from MBCs^31^. It is possible that the advantage we observed with the MF59 adjuvant is due to an early TFH1 rise which leads to more rapid and efficient MBC selection, clonal expansion, and superior antibody quality. Similarly, a MF59-adjuvanted monovalent pandemic H1N1 vaccine also increased the avidity of HA1-specific antibodies when assessed by surface plasmon resonance and ELISA^8^. In our study we observed an increase in anti-H3-HA IgG antibody avidity for A-eIIV recipients, which may be driven by pre-existing HA head-specific MBCs, as there was no increase in the avidity of stalk-specific antibodies.

Antigen-specific CD4^+^ TFH cells are essential for the selection, expansion, and differentiation of memory MBCs into antibody-secreting plasmablasts and plasma cells. Prior studies have detected transient HA-specific circulating TFHs following influenza vaccination^32^ that are clonally related to GC-derived TFH cells^33^. Therefore, we quantified total circulating TFHs as a surrogate for influenza virus-specific TFH response occurring in the lymphoid compartment. We found a positive correlation between the recruitment of TFH1 cells and the increase of day 30 anti-HA antibodies following vaccination with A-eIIV and H-eIIV (Fig. 3e). Similarly, studies in mice have shown that higher antigen dosage^34^ and MF59 adjuvant^7^ increase TFH cell activation. Meanwhile, Herati et al.^16^ reported an age-associated impairment in the helper capacity of ICOS^+^PD-1^+^ TFH cells following S-IIV immunization. Hence, our results suggest that A-eIIV and H-eIIV may overcome impairment of TFH1 cell helper capacity with increased age.

Lifetime exposure to influenza virus drives HA-head predominant antibody responses and stalk antibodies emerge in the absence of HA-head antibodies upon encountering divergent influenza virus strains^35^. Therefore, we assessed each vaccine’s capacity to overcome HA-head immunodominance by the induction of stalk antibodies. We found that A-eIIV induced both G2 and G1-stalk antibodies, whilst H-eIIV boosted only G1-stalk antibodies (Fig. 1g and Supplementary Fig. 3b). Recently, a household transmission study reported that HA-stalk antibodies can act as an independent predictor of protection^36^. Therefore, it is possible that A-eIIV and H-eIIV afford improved protective efficacy through their ability to induce HA-stalk antibodies.

Repeatedly vaccinated individuals have reportedly reduced vaccine effectiveness^37^, antibody^27^, and cellular responses^38^, although blunting effects have not been uniformly shown^39^. We observed that regularly immunized individuals have blunted recruitment of TFH1 cells and corresponding humoral responses. Yet, frequently vaccinated individuals that subsequently received eIIVs also had a trend for increased antibody avidity, and H-eIIV recipients also had improved NK cell-activating antibodies.

Consistent with previous reports, our data showed subclass distribution of HA antibodies consisted mainly of IgG1 and IgG3, whereas stalk antibodies were mainly IgG1^40^. We observed preferential expansion of H3-HA IgG1 above IgG3 in all subjects (Fig. 1f) and an IgG1 driven increase of G2-stalk antibodies in A-eIIV subjects (Supplementary Fig. 2d). IgG3 is considered the most functional subclass due to its strongest affinity for Fc receptors^23^. Meanwhile, aging is associated with impairment of IgG1 production^41^ and this has been hypothesized as a possible cause for lower vaccine efficacy in older adults. Therefore, the boosting of H3-HA in both IgG1 and IgG3 subclasses following vaccination is encouraging. Previously, a comparison between purified influenza virus-specific IgG1 versus IgG3 reported comparable virus neutralization potency^42^, but any distinct subclass advantages for other antibody-mediated effector functions remains undetermined.

Studies in younger and older adults have shown that HA-specific ADCC antibodies are boosted following seasonal vaccination^19,24^. Similarly, we observed increased H3-HA-specific ADCC responses following vaccination in all vaccine groups (Fig. 2a), with the greatest induction of response among H-eIIV recipients (Fig. 2b), while A-eIIV recipients had further boosting of G2 stalk-specific ADCC responses (Fig. 2c, d). Remarkably, boosting of HA-specific and G2-stalk NK cell responses was observed in both HAI responders and nonresponders (Supplementary Fig. 4), suggesting that ADCC responses can be independent markers of vaccine responsiveness, with A-eIIV and H-eIIV conferring distinct advantages.

Polyfunctional T cells are functionally superior to single-cytokine-producing cells. For example, CD8^+^ T cells that coproduce IFN-γ and TNF-α are known to have better cytolytic activity than cells that secrete either cytokine alone^26^. We observed that single-cytokine-producing (IFN-γ^+^, TNF-α^+^, or IL-2^+^) CD8^+^ T cells were readily boosted by A-eIIV and R-eIIV vaccines concurrently with polyfunctional cytokine responses (Fig. 4a and Supplementary Fig. 6b). Importantly, the polyfunctional CD8^+^ T cell IFN-γ^+^TNF-α^+^IL-2^−^ a population that had previously been correlated with reduced disease severity^17^ was significantly expanded by A-eIIV and R-eIIV, potentially indicating an increased T cell protective capacity of these vaccines.

Polyfunctional CD4^+^ T cells produce higher amounts of cytokine per cell, express greater levels of co-stimulation molecules, and have helper capacity potential than single-cytokine-producing cells^43^. Remarkably, we observed that S-IIV recipients had skewed single-cytokine-producing CD8^+^ and CD4^+^ T cell responses compared to eIIVs (Fig. 4f, g). Expression of multiple cytokines requires different activation thresholds and levels of T cell receptor (TCR) signaling for synthesis^44^. Low concentrations of antigen induce predominantly single-cytokine-secreting T cells, whereas multiple-cytokine-secreting T cells with improved function and proliferative abilities emerge following increased antigen concentrations^45^, which may explain for why S-IIV is predisposed toward single-cytokine production and vaccines with a higher antigen content have an advantage over S-IIV. Meanwhile, MF59 adjuvant may have boosted antigen presentation^46^ resulting in increased TCR signaling to drive polyfunctional T cell responses. Recently, Trieu et al.^39^ also reported that repeat annual vaccination with S-IIV boosted CD4^+^ IFN-γ^+^TNF-α^+^IL-2^−^ T cells incrementally over 5 years.

Although R-eIIV contains only purified HA and lacks conserved internal proteins, it stimulated robust multiple-cytokine-producing CD4^+^ T cell subsets that correlated with HAI titers (Fig. 4e). In our initial study, R-eIIV versus S-IIV stimulated the highest rate and level of neutralizing antibodies against the vaccine representative cell-adapted A(H3N2) virus^21^, suggesting that superior microneutralization titers may also be associated with help from high-quality HA-specific CD4^+^ T cells^47^. A mouse study by Nayak et al.^48^, showed that repeated exposure to influenza virus drives CD4^+^ T cell specificity toward conserved internal epitopes and away from non-conserved HA epitopes, resulting in a significant reduction of HA-specific antibodies. It is possible that the purified HA protein composition of R-eIIV results in less competition with pre-existing cross-reactive memory CD4^+^ T cells targeting internal epitopes, thereby re-focusing CD4^+^ T cells to target HA and resulting in higher antibody titers.

We also observed an increase in multiple-cytokine-secreting CD8^+^ T cell responses despite the use of inactivated/nonreplicating vaccines. It is possible that improved CD4^+^ T cell helper activity may amplify CD8^+^ T cell function, for example, through increased co-stimulatory signals or altered interaction between CD4^+^ T cells and antigen-presenting cells (APC). Increased antigen dosage may factor into more efficient cross-presentation, although the intrinsic ability for cross-priming of exogenous antigens is reported to be generally low^49^. It may be possible that improved IgG responses lead to greater antibody-mediated opsonization of viral proteins or immune complex formation, which in turn promote antigen uptake and cross-presentation by APCs leading to improved CD8^+^ T cell responses^50^.

Our study had a number of limitations. Firstly, our experiments used recombinant A(H3N2) A/Switzerland/9715293/2013 HA protein and were mismatched for the vaccine strain A(H3N2) A/Hong Kong/4801/2014, although responses had substantial cross-reactivity in vitro (Supplementary Fig. 1). This meant that we were measuring immunological recall or “back-boosting” to a vaccine strain last used in the 2015/2016 northern hemisphere season. Nevertheless, back-boosting to past seasonal influenza A virus vaccine strains is enhanced by seroconversion toward current season vaccine strains, with the greatest intensity of back-boosting towards antigenically similar strains and among older individuals^51^. Furthermore, approximately 60% of the serum antibody repertoire following vaccination consists of pre-existing clonotypes and not de novo responses toward the vaccine strain^52^. Therefore, our use of the A/Switzerland-lineage H3-2013 HA protein and virus remains relevant. Secondly, we were limited by the number of participants that were able to provide additional blood for PBMC isolation resulting in a limited amount of matched serological and cellular data. This also restricted our ability to stratify data and we have presented pooled datasets when studying vaccination history. Almost two-thirds of our selected participants were previously vaccinated in the prior season and nearly three-quarters had at least one vaccination in the last 5 years. Thus, assessing the full impact of vaccination history and vaccine responsiveness will require further longitudinal analysis in subsequent seasons of our trial. Finally, vaccine immunogenicity is not equivalent to protective efficacy and larger studies are needed to determine immune-mediated protection. As there is yet to be defined, quantitative thresholds for CoPs beyond HAI and MN, we cannot ascertain whether responses we have identified reach protective levels.

In our initial studies, the benefit of enhanced vaccines was evident by the improved magnitude of HAI and IFN-γ^+^ T cell responses against multiple influenza virus strains, particularly for R-eIIV^21^. In this current study, we have shown that all four seasonal influenza vaccines, S-IIV, A-eIIV, H-eIIV, and R-eIIV have comparable magnitude of antibody responses toward H3 HA protein when assessed conventional ELISA assays and differences in vaccine immunogenicity only become apparent when measures of quality are considered. By delving further into antibody quality, we found evidence that A-eIIV generates superior H3-HA antibody avidity and greater G2-stalk antibody titers with effector functions including NK cell activation. Meanwhile, H-eIIV drove the strongest levels of HA-specific NK cell activation which was also extended to individuals with a history of frequent vaccination. Recruitment of high-quality polyfunctional T cells with protective potential against severe influenza disease was also highest in A-eIIV and R-eIIV recipients.

Strategies for universal influenza vaccines aim to generate broadly reactive immune responses beyond neutralizing antibodies against the HA-head domain alone. Hence, it is increasingly important to delineate additional immune correlates of vaccine responsiveness and determine their role in protection to pinpoint pivotal immune players for an all-encompassing universal vaccine. Therefore, as enhanced vaccines are already available, it is important to identify and best engage appropriate immune correlates and effectively utilize these vaccines to achieve optimal protection.

## Methods

### Study approval

Community-dwelling older adults aged 65–82 years were recruited during the 2017/2018 northern hemisphere season in Hong Kong (ClinicalTrials.gov NCT03330132), and all of the patients provided informed consent prior to inclusion in the study. The study protocol was approved by the Institutional Review Board of the University of Hong Kong (UW:16-2014).

### Study design and sampling

Following randomization, a total of 800 donors (200 per group) received either Fluquadri (quadrivalent S-IIV, Sanofi Pasteur, 15 μg HA/per strain), Fluad (MF59-adjuvanted trivalent A-eIIV, Seqirus, 15 μg HA/per strain), Fluzone-High-Dose (trivalent H-eIIV, Sanofi Pasteur, 60μg HA/per strain) or Flublok (recombinant-HA quadrivalent R-eIIV, Protein Sciences, 45 μg HA/per strain). All vaccines contained antigens from A/Michigan/45/2015 (H1N1), A/Hong Kong/4801/2014 (H3N2), and B/Brisbane/60/2008 (Victoria lineage); quadrivalent vaccines contained additional antigens from B/Phuket/3073/2013 (Yamagata lineage). Five years of prior vaccination history for each subject was based on clinical records or self-reporting (Table 1).

Clotted blood was collected immediately before and 30 days post vaccination. Sera was isolated for HAI assays, as previously described^21^. Heparinised blood was collected from a subset of donors immediately before and days 7 and 30 post vaccination (see Table 2) for further humoral and cellular analysis. Plasma was isolated, stored at −80 °C, and heat-inactivated (HI) at 56 °C for 30 min upon testing. Peripheral blood mononuclear cells (PBMC) were isolated by Ficoll-Paque (GE Healthcare) separation using Leucosep tubes (Greiner Bio-one) and cryopreserved in liquid nitrogen. Subjects with complete collection time points were randomly selected for experimental analysis (Table 2). Enzyme-linked immunosorbent assays (ELISA) and ADCC experiments were performed on 20 participants per vaccine group. Intracellular cytokine staining (ICS) of T cells was performed on samples minimum recovery of 2 × 10^5^ live cells during FACS (*n* = 23–24 per vaccine group). TFH and plasmablast staining was performed on fresh blood at the time of collection. However, there were technical issues with immunostaining optimization and complete data across all three time-points was only available for a subset of subjects (*n* = 13–24 per vaccine group). HAI experiments were performed for all study participants who donated additional blood samples for plasma and PBMC isolation (*n* = 23–37 per vaccine group), as previously described^21^.

### Hemagglutination antibody inhibition assay

The HAI assay was carried out as described previously^21^. Briefly, RDE-treated and HI sera were serially titrated in phosphate-buffered saline (PBS) and incubated with four hemagglutinating units of egg-propagated A/Hong Kong/4801/2014 (H3N2) antigen for 1 h at room temperature and then with 0.5% turkey red blood cells for 30 min at room temperature. HAI titers was recorded as the reciprocal of the highest sera dilution that inhibited hemagglutination.

### Enzyme-linked immunosorbent assay

Plates (Nunc MaxiSorp, Thermofisher Scientific) were coated with one representative influenza virus protein at a time. Plates were coated with either 1 μg/ml of purified baculovirus-expressed HA protein from A/Switzerland/9715293/2013 (H3-2013), nucleoprotein (NP) from A/Switzerland/9715293/2013, HEK293-expressed HA protein from A/California/07/09 (H1-2009), HEK293-expressed neuraminidase (NA) protein from A/Hong Kong/4801/2014 (N2-2014), A/Michigan/45/2015 (N1-2015) (SinoBiological), *Escherichia coli* expressed HA-stalk proteins from A/Hong Kong/1/1968 (G2-stalk) or A/Puerto Rico/8/1934 (G1-stalk)^53^ (from Raghavan Varadarajan, Indian Institute of Science). Plates were rinsed, blocked with 1% fetal bovine serum (FBS) in PBS, incubated with 1:100 HI plasma diluted in 0.05% Tween-20/0.1% FBS in PBS for 2 h then rinsed again, and incubated with detection antibodies for 2 h using IgG-HRP (1:5000, G8-185; BD), IgG1-HRP (1:2000, 4E3), IgG2-HRP (1:1500, 31-7-4) IgG3-HRP (1:1500, HP6050) or IgG4-HRP (1:4000 HP6025) (IgG1/2/3/4 detection antibodies from SouthernBiotech). For detection of high-avidity antibodies, an additional 8 M urea wash step (3× 5 min) was performed on a separate plate before the addition of anti-IgG-HRP detection antibody^54^. The color was developed with equal amounts of stabilized hydrogen peroxide and tetramethylbenzidine (R&D systems) for 20 min, stopped with 2N sulfuric acid and absorbance values were recorded at 450 nm on a spectrophotometer (Tecan Life Sciences). The avidity index was calculated as a percentage of the optical density with urea-treatment versus without treatment. A 4-parameter logistic regression (4PL) method was used to interpolate absorbance values from a standard curve generated from purified human IgG (I4506, Sigma Aldrich).

### Antibody-mediated NK cell activation

Plates were coated with 4μg/ml of H3-2013 or G2-stalk protein, rinsed and blocked, incubated with 1:400 HI plasma diluted in PBS for 2 h, then incubated with NK-92-FcRγIIIA-bearing cells (Fox Chase Institute for Cancer Research) at 37 °C for 5 h. Cells were then stained using anti-human CD56-PE (5.1H11) and CD107a-APC (H4A3) (Biolegend), fixed with 4% paraformaldehyde (PFA) and acquired by flow cytometry (AttuneNxT). Purified anti-human LEAF CD16 antibody (3G8) and irrelevant HIV-1 gp120 protein were used as positive and negative controls, respectively. Responses were subtracted for background and normalized to the percentage of maximum positive controls.

### Immunostaining of TFH cells and plasmablasts

Whole blood samples were stained with indicated antibodies (all Biolegend and clone used). The TFH panel contained: anti-human CD3-PE/Dazzle 594 (UCHT1), CD4-AlexaFluor700 (SK3), CXCR5-PerCPCy5.5 (J252D4), CD45RA-FITC (HI100), CCR6-BV605 (G034E3), CXCR3-APC (G025H7), PD-1-BV711 (EH12.2H7), and ICOS-PE (C398.4A). A separate plasmablast panel contained: CD19-BV510 (HIB19), CD27-FITC (M-T271), and CD38-BV421(HIT2). Cells were acquired by flow cytometry (BD LSRFortessa).

### Intracellular cytokine staining

Cryopreserved PBMCs were thawed and restimulated with a multiplicity of infection (MOI) 4 of UV-irradiated A/HK/4851970/2014 virus (H3N2 Switzerland lineage), PMA/ionomycin or RPMI alone for 6 h at 37 °C. Brefeldin A and monensin were then overnight. Cells were stained with Zombie-NIR (Biolegend) followed by anti-human CD3-PE/Dazzle 594 (UCHT1), CD4-BV605 (OKT4), CD8-AlexaFluor700 (SK1), CD107a-PacificBlue (H4A3), CCR5-PE (J418F1), CCR7-PerCP/Cy5.5 (G043H7), and CD45RA-APC (HI100) and a dump channel containing CD19-BV510 (HIB19), CD56-BV510 (HCD56), and CD14-BV510 (M5E2). Cells were then permeabilized and fixed (BD Cytofix/cytoperm) and further stained for anti-human IFN-γ-FITC (4S.B3), IL-2-PECy7 (MQ1-17H12), and TNFα-BV711 (MAb11). Stained cells were acquired via flow cytometry (AttuneNxT). Background no virus responses were subtracted from each sample.

### Statistical analysis

Statistical analysis was performed on Prism 7 (Graphpad). For multiple-group comparisons, a Friedman (paired) or Kruskal–Wallis (unpaired) test, followed by the Dunn-Bonferroni post-hoc test was used. For two-ground comparison, the Wilcoxon signed-rank test (paired) or Mann–Whitney *t* test (unpaired) was used. Correlations were performed using Spearman’s test. Differences in baseline characteristics were detected with the chi-square test. Adjusted *p* values < 0.05 were considered statistically significant.

### Reporting summary

Further information on research design is available in the Nature Research Reporting Summary linked to this article.

## Supplementary information

Supplementary figures

Reporting Summary

## Data Availability

The data that support the findings of this study are available from the corresponding author upon request.
