# Supplementary material for: Immunogenicity of standard, high-dose, MF59-adjuvanted, and recombinant-HA seasonal influenza vaccination in older adults
Source: NPJ Vaccines. 2021 Feb 16;6:25. doi: 10.1038/s41541-021-00289-5 (PMC7886864; doi:10.1038/s41541-021-00289-5)
Supplement: Supplementary file 1 — Supplementary figures [file 41541_2021_289_MOESM1_ESM.pdf]

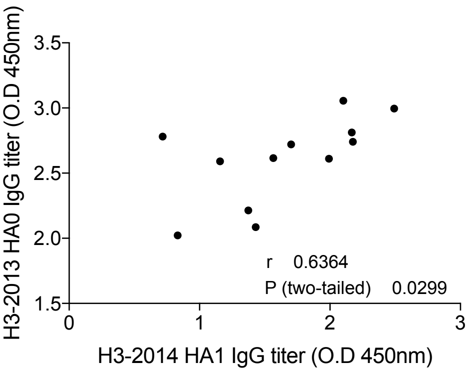

Supplementary Figure 1. Positive correlation between H3-2013 HA0 and H3-2014 HA1 specific IgG antibodies. IgG antibody responses against both proteins were measured by ELISA and Spearman's correlation performed.

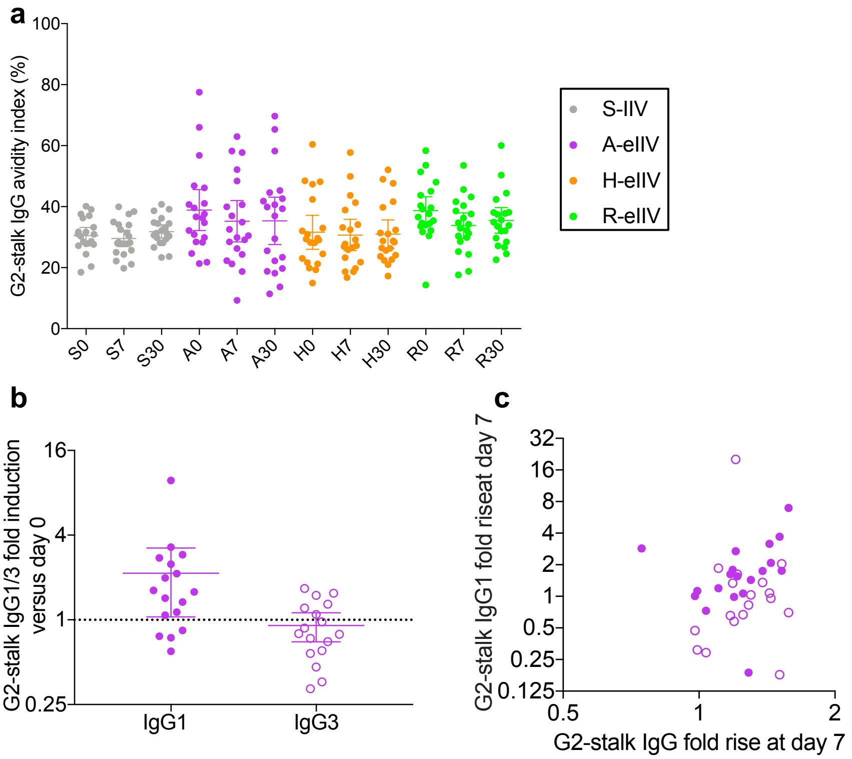

	R	p-value
IgG1	0.48	0.04
IgG3	0.26	0.29

Supplementary Figure 2. Avidity and subclass distribution of G2-stalk IgG antibodies. (a) Proportion of high-avidity G2-stalk-specific IgG antibodies relative to total G2-stalk-specific IgG. **(b)** G2-stalk-specific IgG1/3 subclass distribution and **(c)** Spearman's correlation with total G2-stalk-specific IgG.

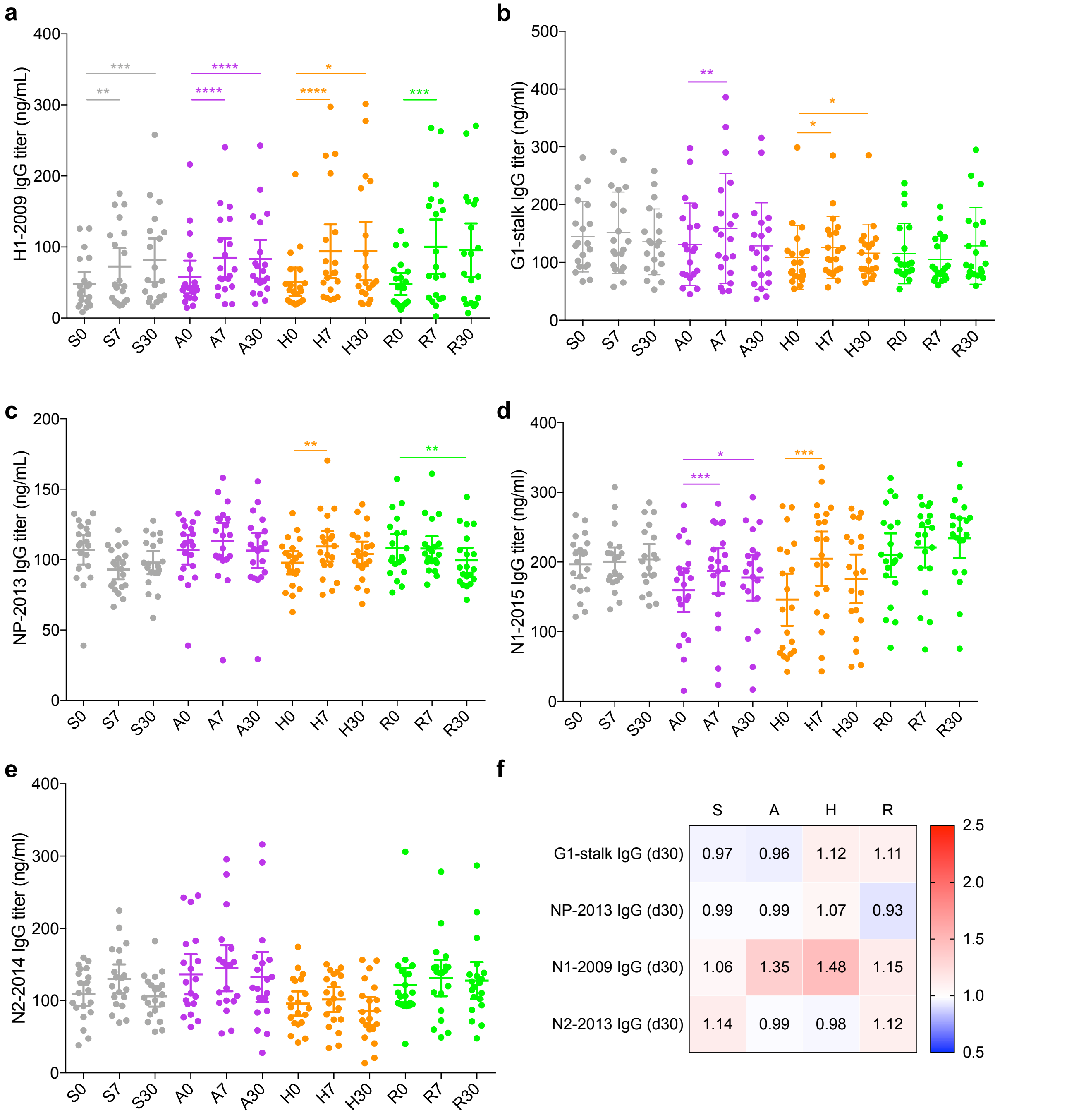

Supplementary Figure 3. Antibody responses to additional vaccine antigens. IgG antibody titers against **(a)** H1-2009 HA, **(b)** G1-stalk, **(c)** NP, **(d)** N1-2015 NA and **(e)** N2-2014 NA and **(f)** summary heat map depicting mean fold induction of responses from (b-e). Data represented as mean (n=20 per vaccine group) with 95%CI; each dot represents a single individual. Friedman's test for multiple-group comparisons was performed. *p<0.05, **p<0.01, ***p<0.001, ****p<0.0001.

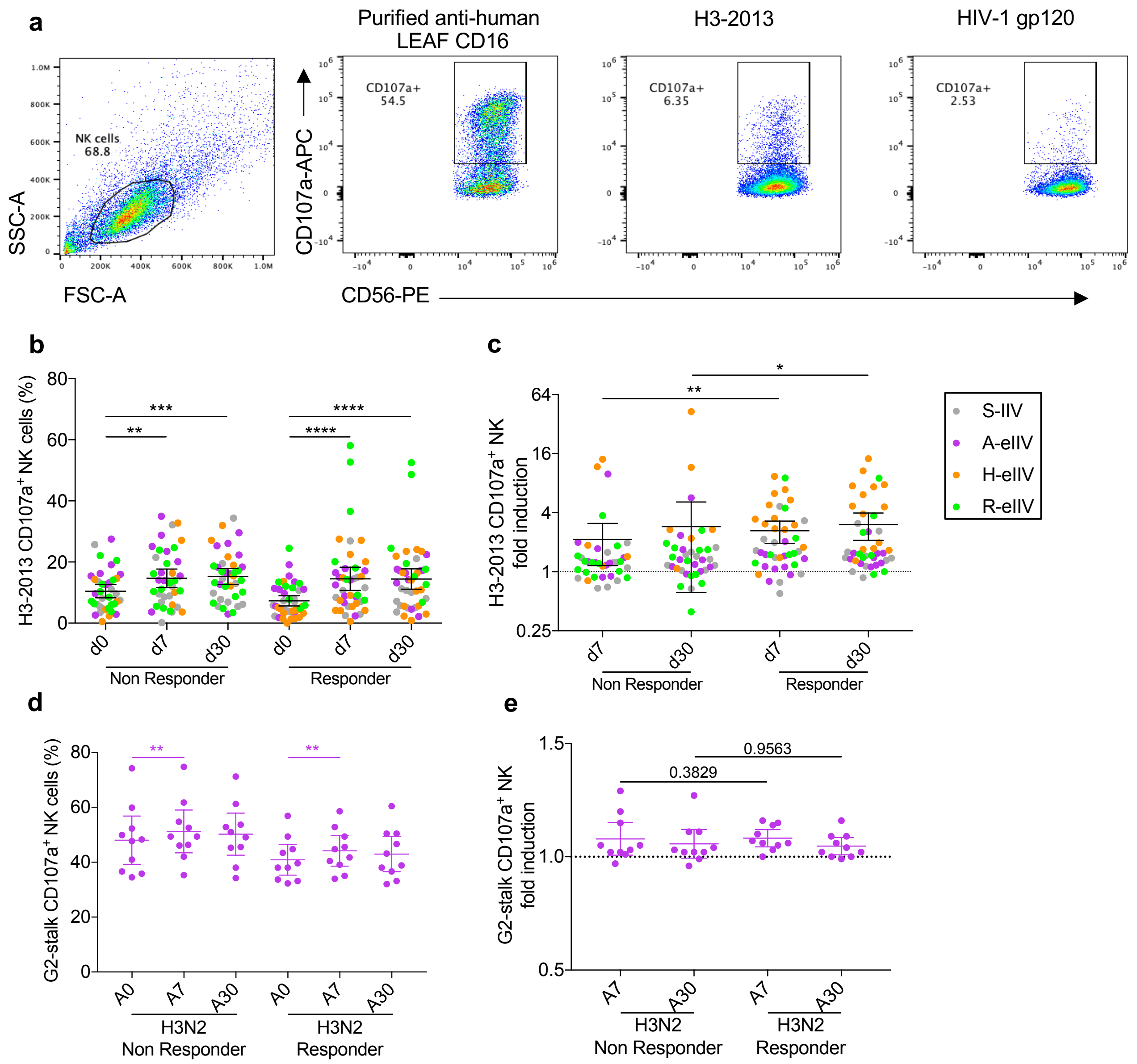

Supplementary Figure 4. HA and stalk-specific antibody-mediated NK cell activation occurs in the absence of neutralizing antibody responses following seasonal influenza vaccination. **(a)** Gating strategy for identification of activated NK cells expressing CD107a degranulation marker, and ADCC responses against anti-human CD16 (positive control), H3-2013 HA, HIV-gp120 (negative control) are depicted. **(b)** Frequency of H3-2013 HA-specific CD107a^{+} NK cells and **(c)** fold induction of response separated by HAI seroconversion status. **(d)** Frequency of G2-stalk-specific CD107a^{+} NK cells and **(e)** fold induction separated by HAI seroconversion status. Data represented as mean with 95%CI; each dot represents a single individual (n=20 per vaccine group). Friedman's test for multiple-group and Mann-Whitney for two-group comparisons was performed. *p<0.05, **p<0.01, ***p<0.001.

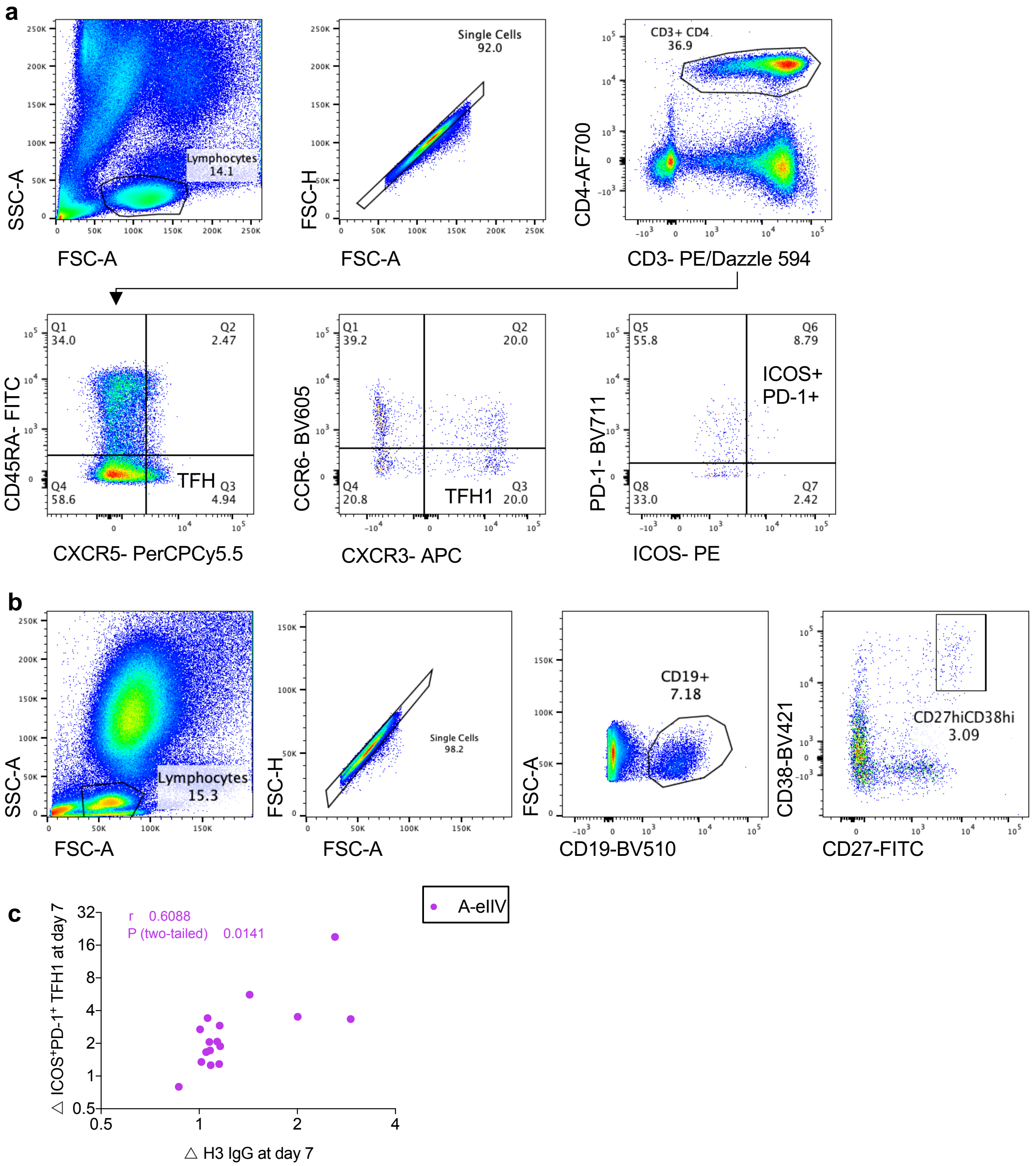

Supplementary Figure 5. Early induction of activated type-1 TFH cells is associated with H3 HA-specific IgG response in A-eIIV recipients. (a) FACS gating strategy for T follicular helper (TFH) cells. Circulating memory TFH cells were defined as $CD4^{+}CXCR5^{+}CD45RA^{-}$. The type-1 TFH subset were defined by expression of $CXCR3^{+}CCR6^{-}$. Co-expression of $ICOS^{+}$ and $PD\text{-}1^{+}$ were defined as activation markers. **(b)** Gating strategy for plasmablasts by expression of activation markers $CD27^{hi}CD38^{hi}$ within the $CD19^{+}$ B cell population. **(c)** Correlation between the fold induction of $ICOS^{+}PD\text{-}1^{+}$ TFH1 cells and H3-2013 HA-specific IgG at day 7 in A-eIIV recipients.

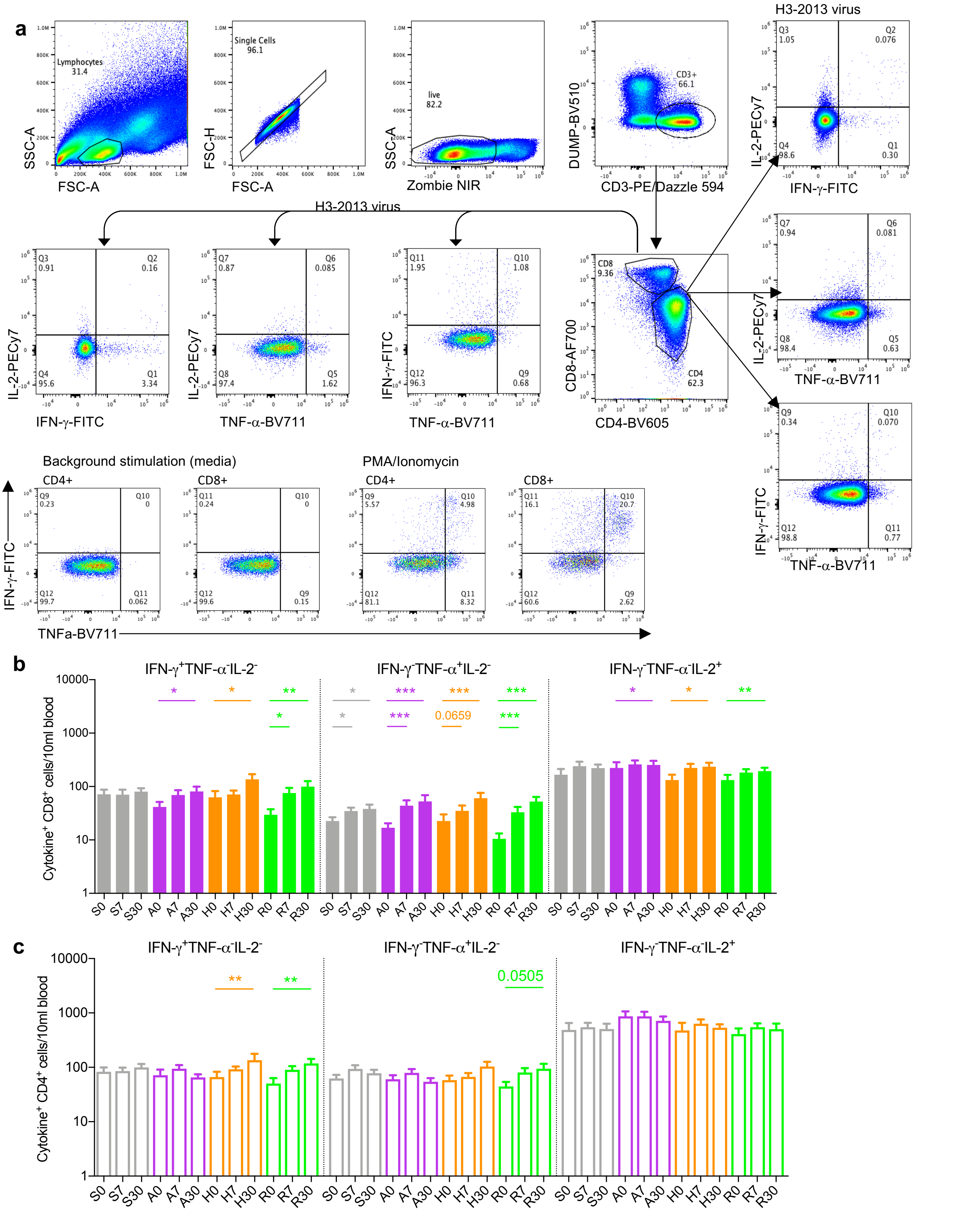

Supplementary Figure 6. H3N2-2013-virus-specific single-cytokine-producing T cells are expanded by enhanced influenza vaccination. (a) Gating Strategy for identification of cytokine-producing T cells. T cells were identified by expression of $CD3^+$ and separated into $CD4^+$ and $CD8^+$ subsets. $CD19^+$ B cells, $CD14^+$ monocytes and $CD56^+$ NK cells were excluded using a DUMP channel. Within each $CD8^+$ and $CD4^+$ population, single, double and triple cytokine-producing IFN-γ, TNF-α and IL-2 subsets were identified in non-overlapping permutations. The magnitude of single cytokine-producing **(b)** $CD8^+$ and **(c)** $CD4^+$ T cell subsets at days 0, 7 and 30 post-vaccination. Data represented as mean (n=22-24 per vaccine group) with SEM. Friedman's test. $^*p<0.05$, $^{**}p<0.01$, $^{***}p<0.001$.

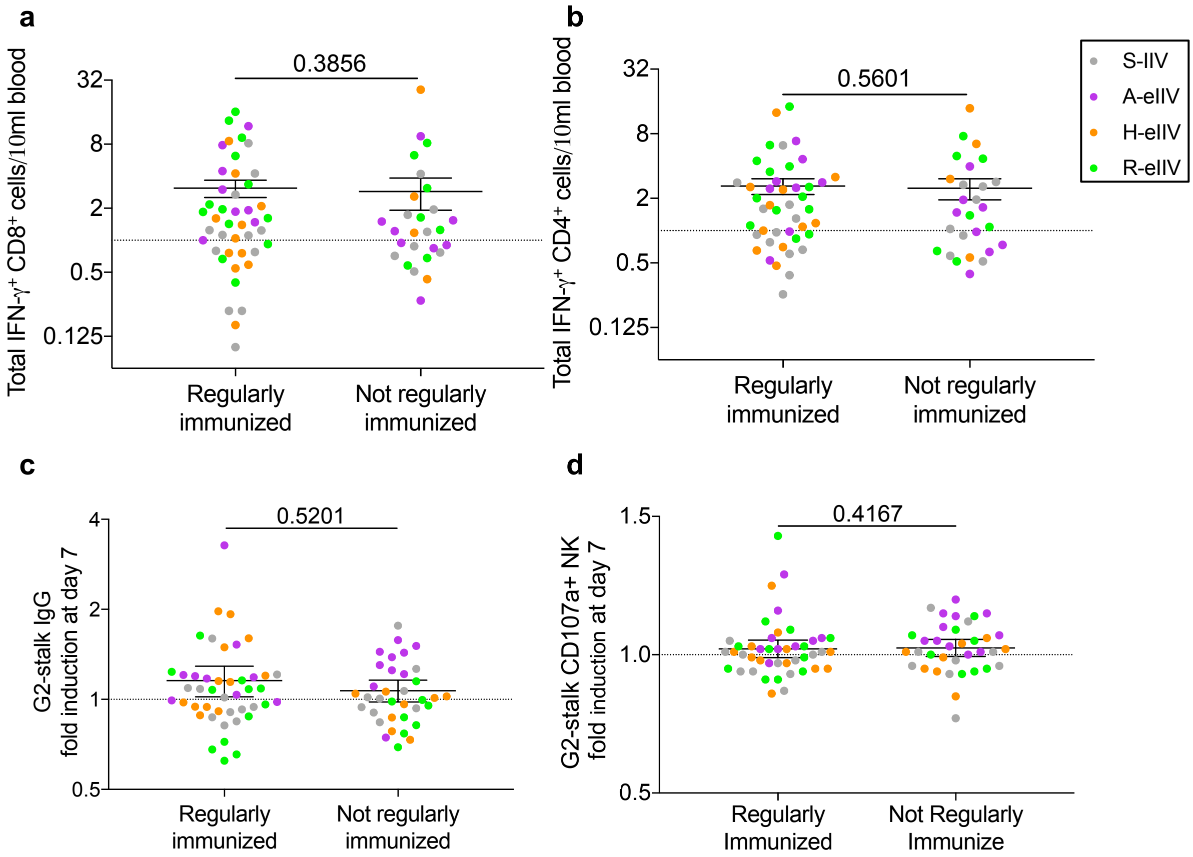

Supplementary Figure 7. Prior vaccination does not affect the magnitude of G2-stalk-specific antibody response. Fold induction of total H3N2-2013-virus-specific IFN-γ^+ $CD8^+$ **(a)** and $CD4^+$ **(b)** T cells (n=22-24 per vaccine group), **(c)** G2-stalk-specific IgG antibodies and **(d)** $CD107a^+$ NK cells (n=20 per vaccine group) separated by frequency of prior vaccination, significance by Mann-Whitney t-test. Data represented as mean with 95%CI; each dot represents a single individual.
