# Supplementary material for: Immunogenicity of standard, high-dose, MF59-adjuvanted, and recombinant-HA seasonal influenza vaccination in older adults
Source: NPJ Vaccines. 2021 Feb 16;6:25. doi: 10.1038/s41541-021-00289-5 (PMC7886864; doi:10.1038/s41541-021-00289-5)
Supplement: Supplementary file 2 — Reporting Summary [file 41541_2021_289_MOESM2_ESM.pdf]

nature research

Corresponding author(s): Valkenburg, NPJVACCINES-00974-T

Last updated by author(s): Nov 9, 2020

Reporting Summary

Nature Research wishes to improve the reproducibility of the work that we publish. This form provides structure for consistency and transparency in reporting. For further information on Nature Research policies, see our Editorial Policies and the Editorial Policy Checklist.

Statistics

For all statistical analyses, confirm that the following items are present in the figure legend, table legend, main text, or Methods section.

n/a	Confirmed	
☐	☒	The exact sample size (*n*) for each experimental group/condition, given as a discrete number and unit of measurement
☐	☒	A statement on whether measurements were taken from distinct samples or whether the same sample was measured repeatedly
☐	☒	The statistical test(s) used AND whether they are one- or two-sided<br>*Only common tests should be described solely by name; describe more complex techniques in the Methods section.*
☐	☒	A description of all covariates tested
☐	☒	A description of any assumptions or corrections, such as tests of normality and adjustment for multiple comparisons
☐	☒	A full description of the statistical parameters including central tendency (e.g. means) or other basic estimates (e.g. regression coefficient) AND variation (e.g. standard deviation) or associated estimates of uncertainty (e.g. confidence intervals)
☐	☒	For null hypothesis testing, the test statistic (e.g. *F*, *t*, *r*) with confidence intervals, effect sizes, degrees of freedom and *P* value noted<br>*Give P values as exact values whenever suitable.*
☒	☐	For Bayesian analysis, information on the choice of priors and Markov chain Monte Carlo settings
☒	☐	For hierarchical and complex designs, identification of the appropriate level for tests and full reporting of outcomes
☐	☒	Estimates of effect sizes (e.g. Cohen's *d*, Pearson's *r*), indicating how they were calculated

Our web collection on statistics for biologists contains articles on many of the points above.

Software and code

Policy information about availability of computer code

Data collection	Flow cytometer Invitrogen Attune NxT, spectrophotometer plate reader (Tecan Life Sciences)
Data analysis	Graphpad Prism version 7, Microsoft Excel, FlowJo version 10

For manuscripts utilizing custom algorithms or software that are central to the research but not yet described in published literature, software must be made available to editors and reviewers. We strongly encourage code deposition in a community repository (e.g. GitHub). See the Nature Research guidelines for submitting code & software for further information.

Data

Policy information about availability of data

All manuscripts must include a data availability statement. This statement should provide the following information, where applicable:

- Accession codes, unique identifiers, or web links for publicly available datasets
- A list of figures that have associated raw data
- A description of any restrictions on data availability

The data that support the findings of this study are available from the corresponding author upon request.

Field-specific reporting

Please select the one below that is the best fit for your research. If you are not sure, read the appropriate sections before making your selection.

☒ Life sciences ☐ Behavioural & social sciences ☐ Ecological, evolutionary & environmental sciences

For a reference copy of the document with all sections, see nature.com/documents/nr-reporting-summary-flat.pdf

Life sciences study design

All studies must disclose on these points even when the disclosure is negative.

Sample size	Enzyme-linked immunosorbent assays (ELISA) and ADCC experiments were performed on 20 participants per vaccine group. Intracellular cytokine staining (ICS) of T cells was performed on samples minimum recovery of 2x105 live cells during FACS (n=23-24 per vaccine group). TFH and plasmablast staining was performed on fresh blood at the time of collection. However, there were technical issues with immunostaining optimization and complete data across all three time-points was only available for a subset of subjects (n=13-24 per vaccine group). HAI experiments were performed for all study participants who donated additional blood samples for plasma and PBMC isolation (n=23-37 per vaccine group), as previously described. (Cowling CID 2019)
Data exclusions	Selected samples were excluded for the T cell analysis based on cell counts below 200,000 live events.
Replication	Experiments were performed twice with the same internal controls.
Randomization	Participants were randomized for vaccination group at enrollment.
Blinding	Participants and field nurses were blinded at enrollment. Investigators were not blinded to group allocation or time point at analysis.

Reporting for specific materials, systems and methods

We require information from authors about some types of materials, experimental systems and methods used in many studies. Here, indicate whether each material, system or method listed is relevant to your study. If you are not sure if a list item applies to your research, read the appropriate section before selecting a response.

Materials & experimental systems

n/a	Involved in the study	
☐	☒	Antibodies
☐	☒	Eukaryotic cell lines
☒	☐	Palaeontology and archaeology
☒	☐	Animals and other organisms
☐	☒	Human research participants
☐	☒	Clinical data
☒	☐	Dual use research of concern

Methods

n/a	Involved in the study	
☒	☐	ChIP-seq
☐	☒	Flow cytometry
☒	☐	MRI-based neuroimaging

Antibodies

Antibodies used	anti-human CD3-PE/Dazzle 594 (UCHT1), CD4-AF700 (SK3), CXCR5-PerCPCy5.5 (J252D4), CD45RA-FITC (HI100), CCR6-BV605 (G034E3), CXCR3-APC (G025H7), PD-1-BV711 (EH12.2H7), ICOS-PE (C398.4A), CD19-BV510 (HIB19), CD27-FITC (M-T271), CD38-BV421(HIT2)CD56-PE (5.1H11), CD107a-APC (H4A3), LEAF CD16 antibody (3G8), CD4-BV605 (OKT4), CD8-AlexaFluor700 (SK1), CD107a-PacificBlue (H4A3), CCR5-PE (J418F1), CCR7-PerCP/Cy5.5 (G043H7), CD45RA-APC (HI100), CD19-BV510 (HIB19), CD56-BV510 (HCD56) and CD14-BV510 (M5E2), IFN-g-FITC (4S.B3), IL-2-PECy7 (MQ1-17H12), TNFa-BV711 (MAb11). (Biolegend)<br><br>IgG-HRP (1:5000, G8-185; BD)<br>IgG1-HRP (1:2000, 4E3), IgG2-HRP (1:1500, 31-7-4) IgG3-HRP (1:1500, HP6050) or IgG4-HRP (1:4000 HP6025) (IgG1/2/3/4 detection antibodies from SouthernBiotech)
Validation	For Flow cytometry antibodies were assessed by single color staining at the time of acquistion, and titrations on PBMCs to determine optimal staining concentration.<br>For ELISA antibodies were titrated to determine optimal concentrations used plus an IgG standard.

Eukaryotic cell lines

Policy information about cell lines

Cell line source(s)	NK-92 cells, Fox Chase Cancer center

Authentication	CD16 staining
Mycoplasma contamination	Cell lines tested for mycoplasma
Commonly misidentified lines (See ICLAC register)	Not applicable

Human research participants

Policy information about studies involving human research participants

Population characteristics	As described Table 1.
Recruitment	As previously described. Cowling, B. J. et al. Comparative Immunogenicity of Several Enhanced Influenza Vaccine Options for Older Adults: A Randomized, Controlled Trial. Clin Infect Dis, doi:10.1093/cid/ciz1034 (2019)
Ethics oversight	The study protocol was approved by the Institutional Review Board of the University of Hong Kong (UW:16-2014).

Note that full information on the approval of the study protocol must also be provided in the manuscript.

Clinical data

Policy information about clinical studies

All manuscripts should comply with the ICMJE guidelines for publication of clinical research and a completed CONSORT checklist must be included with all submissions.

Clinical trial registration	NCT03330132
Study protocol	Cowling, B. J. et al. Comparative Immunogenicity of Several Enhanced Influenza Vaccine Options for Older Adults: A Randomized, Controlled Trial. Clin Infect Dis, doi:10.1093/cid/ciz1034 (2019)
Data collection	Participants were recruited from October 2017 to January 2018 in Hong Kong
Outcomes	Immunogenicity trial

Flow Cytometry

Plots

Confirm that:

- [x] The axis labels state the marker and fluorochrome used (e.g. CD4-FITC).
- [x] The axis scales are clearly visible. Include numbers along axes only for bottom left plot of group (a 'group' is an analysis of identical markers).
- [x] All plots are contour plots with outliers or pseudocolor plots.
- [x] A numerical value for number of cells or percentage (with statistics) is provided.

Methodology

Sample preparation	As described, ficoll paque density centrifugation, cryo perservation and thawing for stimulation with UV inactviated virus for cytokine production.
Instrument	Invitrogen Attune NxT
Software	FlowJo version 10
Cell population abundance	A minimum of 200,000 live cells was acquired for T cell analysis, and 5,000 live cells for NK92 ADCC assays.
Gating strategy	As described Figure S4, S5, S6.

- [x] Tick this box to confirm that a figure exemplifying the gating strategy is provided in the Supplementary Information.
